# Individual Response Variation in the Effects of Weight Loss and Exercise on Insulin Sensitivity and Cardiometabolic Risk in Older Adults

**DOI:** 10.3389/fendo.2020.00632

**Published:** 2020-09-10

**Authors:** Andrea M. Brennan, Robert A. Standley, Fanchao Yi, Elvis A. Carnero, Lauren M. Sparks, Bret H. Goodpaster

**Affiliations:** Translational Research Institute, AdventHealth Research Institute, Orlando, FL, United States

**Keywords:** individual variability, weight loss, exercise, insulin sensitivity, response, cardiometabolic risk, older adults

## Abstract

Weight loss induced by decreased energy intake (diet) or exercise generally has favorable effects on insulin sensitivity and cardiometabolic risk. The variation in these responses to diet-induced weight loss with or without exercise, particularly in older obese adults, is less clear. The objectives of our study were to (1) examine the effect of weight loss with or without exercise on the variability of responses in insulin sensitivity and cardiometabolic risk factors and (2) to explore whether baseline phenotypic characteristics are associated with response. Sedentary older obese (BMI 36.3 ± 5.0 kg/m^2^) adults (68.6 ± 4.7 years) were randomized to one of 3 groups: health education control (HED); diet-induced weight loss (WL); or weight loss and exercise (WL + EX) for 6 months. Composite Z-scores were calculated for changes in insulin sensitivity (C_IS: rate of glucose disposal/insulin at steady state during hyperinsulinemic euglycemic clamp, HOMA-IR, and HbA1C) and cardiometabolic risk (C_CMR: waist circumference, triglycerides, and fasting glucose). Baseline measures included body composition (MRI), cardiorespiratory fitness, *in vivo* mitochondrial function (ATPmax; P-MRS), and muscle fiber type. WL + EX groups had a greater proportion of High Responders in both C_IS and C_CMR compared to HED and WL only (all *p* < 0.05). Pre-intervention measures of insulin (*r* = 0.60) and HOMA-IR (*r* = 0.56) were associated with change in insulin sensitivity (C_IS) in the WL group (*p* < 0.05). Pre-intervention measures of glucose (*r* = 0.55), triglycerides (*r* = 0.53), and VLDL (*r* = 0.53) were associated with change in cardiometabolic risk (C_CMR) in the WL group (*p* < 0.05), whereas triglycerides (*r* = 0.59) and VLDL (*r* = 0.59) were associated with C_CMR (all *p* < 0.05) in WL + EX. Thus, the addition of exercise to diet-induced weight loss increases the proportion of older obese adults who improve insulin sensitivity and cardiometabolic risk. Additionally, individuals with poorer metabolic status are more likely to experience greater improvements in cardiometabolic risk during weight loss with or without exercise.

## Introduction

Aging is associated with increased adiposity, insulin resistance and a higher prevalence of cardiometabolic disease (1). Current evidence suggests that weight loss induced by decreased energy intake or increased energy expenditure improves insulin sensitivity and cardiometabolic risk factors in older adults (2). While limited data exists on the effect of diet-induced weight loss alone on individual variability in cardiometabolic and glycemic control outcomes, several groups have observed substantial heterogeneity in individual responses to exercise-induced weight loss with or without dietary changes. The first observations of interindividual variability in glycemic control indices following exercise stem from the HERITAGE Family Study, wherein ~600 healthy sedentary individuals completed a 20-week supervised training intervention (3). Although there were statistically significant increases with training in insulin sensitivity measured by intravenous glucose tolerance test (IVGTT) at the group level, the authors noted that ~42% of participants showed no change or an adverse response. Similar response variation in HOMA-IR (4), HbA1C, fasting glucose, and 2-h oral glucose tolerance test (OGTT) glucose (5) was observed after both high intensity interval training (4) and continuous aerobic exercise (5). An important limitation of the preceding study designs is lack of a time-matched control group which abrogates the ability to account for response variation due to technical error and/or biological fluctuations (6, 7). Additionally, whether diet-induced weight loss with or without exercise differentially improves response variation in glycemic control and cardiometabolic risk is unknown, albeit potentially important for clinicians providing personalized lifestyle counsel.

While there is little doubt that response variation to exercise interventions exists, the characteristics that distinguish those who do and do not respond favorably are unclear. Multiple observations have suggested that baseline glycemic control is a key factor that predicts the magnitude of an individual's response for glycemic control outcomes. Evidence from a 3 month aerobic exercise training intervention in older obese adults illustrated an inverse relationship between baseline fasting glucose and change in fasting glucose (8), suggesting that those with higher baseline glycemia had a blunted response to exercise. Similarly, following a 3–4 month exercise training intervention in 105 individuals with prediabetes or type 2 diabetes mellitus (T2DM), Solomon et al. (5) observed a non-linear U-shaped relationship between baseline HbA1c and change in HbA1C, postulating that individuals with relatively controlled hyperglycemia respond well to training, while individuals with poor glycemic control have blunted improvements or even deteriorations. However, not all findings are consistent with the aforementioned results. Recent findings wherein 285 participants aged 18–75 years participated in a 12-week lifestyle intervention including both dietary and exercise guidance suggested that those who experienced greater improvement in glucose tolerance presented with higher baseline weight, visceral fat, fasting glucose, and triglyceride concentration compared to those who did not respond (9). Thus, it is unclear whether and how baseline phenotypes influence insulin sensitivity responses to diet and exercise-induced weight loss, and response likely depends on multiple factors.

Several gaps in our current knowledge exist concerning response variation in weight loss: (1) examination of the independent (and potentially additive) effect of exercise, particularly in higher risk older obese adults; (2) the use of a control group to examine technical and/or biological changes; and (3) a more wholistic classification of the clinically meaningful outcome that includes a cluster of interrelated responses. Thus, the objectives of our study are 2-fold: (1) to examine the effect of weight loss with or without exercise on the range of responses in insulin sensitivity and cardiometabolic risk factors in a vulnerable older obese population at risk for chronic disease; (2) to perform a comprehensive assessment of the relationships between baseline clinical, metabolic, and skeletal muscle traits and changes in response to weight loss with or without exercise. We conducted a randomized controlled trial to examine the effect of energy restriction-induced weight loss with or without exercise on insulin sensitivity and skeletal muscle function in older obese adults, providing a unique opportunity to address these aims. We hypothesize that the addition of exercise to diet-induced weight loss will increase the number of individuals who respond favorably to intervention, based on improvements in insulin sensitivity and cardiometabolic risk. Our findings may provide mechanistic and clinical insight into response variation to weight loss interventions in this vulnerable population.

## Materials and Methods

### Participants

The participants included in this analysis were a subset of participants enrolled in a larger RCT (unpublished; NCT number: NCT02230839). We conducted a single site, 6-month randomized controlled trial with a parallel group design between 2012 and 2017. The trial operations began at the University of Pittsburgh and subsequently moved to AdventHealth Translational Research Institute (AH TRI) upon re-appointment of the Primary Investigator. Eighty-six older (60–80 years of age), sedentary (≤ 1 continuous exercise session/week) men and women with obesity (BMI ≥ 30 kg/m^2^) were randomized into one of three treatments: Control (HED; health education); energy restriction-induced weight loss (WL; 10% weight loss), and weight loss with exercise (WL + EX; progressive, moderate intensity supervised exercise sessions). All participants provided informed consent prior to participation and the protocols used in the original investigation and this secondary analysis were approved by both University of Pittsburgh Research Ethics Board and Institutional Review Board of AdventHealth. Participants from the original trial were excluded if they did not have both pre- and post- outcome data (*n* = 25) which resulted in a study sample of 61 participants: HED, *n* = 20; WL, *n* = 21; WL + EX, *n* = 20.

#### Health Education (HED) Group

Participants randomized to the HED group received bi-weekly in-person general health education group sessions for the 6-month study duration, including informational seminars on medication and type 2 diabetes management. Each session lasted ~1 h. However, they were not given specific exercise or dietary education/prescription.

#### Energy Restriction-Induced Weight Loss (WL) Group

The goal of the WL intervention was to produce a weight loss of 10% of baseline body weight. Using the Harris-Benedict equation corrected for the activity factor, a reduction of 500–1,000 kcal/day based on baseline body weight was prescribed in addition to a low-fat (<30% of kilocalories from fat) diet. Participants met individually with the Registered Dietitian and/or designated staff weekly to record body weight and receive dietary prescription (~1 h). To eliminate the confounding effects of acute caloric restriction on insulin sensitivity, the dietitian aimed to keep participant weights stable during the last 2 weeks of intervention.

#### Weight Loss and Exercise (WL + EX) Group

Participants completed a progressive 6-month exercise training program, 4–5 days per week, 45 min per session (180 min per week) consisting of mostly walking (both outside and on an indoor treadmill) and the option to include stationary cycling, elliptical and rowing machines. All indoor exercise was supervised by a trained monitor; aerobic exercise performed outdoors was not supervised. Beginning at week 8, participants also performed 2, non-consecutive resistance exercise sessions per week, 30 min per session, focused on major muscle groups using resistance exercise machines. Aerobic exercise was performed at 50–80% HR_reserve_. The resistance exercises were performed at the highest weight the participant could achieve for the given number of reps (10–12) with proper form. When the participant reached 3 × 12 reps, we increased the weight and reduced the reps. Blood pressure and heart rate were measured for participant safety prior to each exercise session, in addition to weekly body weight. Participants in the WL + EX group also met with the Registered Dietitian and/or designated staff and received the same dietary instruction as the WL group.

### Outcomes

#### Body Composition

Weight and height were measured pre- and post- intervention, and BMI was calculated. Waist circumference was measured using the Gulick II tape measure directly on the skin. Fat mass and fat-free mass were determined by dual-energy X-ray absorptiometry (DXA) using a GE Lunar (GE Healthcare, UK).

Additionally, abdominal and thigh adipose tissue (AT) and muscle volume was measured by MRI at baseline and following treatment on a 3 Tesla magnet (Philips Acheiva) at AH TRI. The MRI scan was performed at the mid-point of the femur to quantify thigh muscle cross-sectional area, subcutaneous, and intermuscular AT (IMAT). For abdominal AT images, high resolution axial images were taken of the entire abdomen to quantify abdominal subcutaneous and visceral AT volume. Resultant images were analyzed using Analyze 11.0 (Biomedical Imaging Resource, Mayo Clinic, Rochester, MN) to segment AT and muscle depots and measure volume.

#### Cardiorespiratory Fitness and *in vivo* Mitochondrial Function

A VO_2max_ graded exercise test was performed by an exercise physiologist on the cycle ergometer using open circuit indirect calorimetry. Following a standardized warm-up, participants exercised at a moderate intensity with the workload (resistance) increased gradually until they reached volitional fatigue.

*In vivo* muscle mitochondrial function (ATPmax) was calculated using the PCR recovery time constant (τ) and the PCr level in oxygenated muscle at rest in the *vastus lateralis* using phosphorus (^31^P) magnetic resonance spectroscopy on the 3-T magnet as previously described (10).

#### Insulin Sensitivity

Insulin sensitivity was measured using the hyperinsulinemic-euglycemic clamp. Participants arrived at the research facility prior to the clamp procedure, consumed a standard American meal, and stayed overnight in the metabolic ward. After an overnight fast, an intravenous catheter was placed in the antecubital vein for subsequent insulin and glucose infusions and for stable isotope infusions to measure insulin sensitivity. A primed constant infusion of [6,6-2H2] ran throughout the clamp procedure. An additional catheter was placed in the heated hand vein in the contralateral arm to attain arterialized blood samples for blood glucose determination and for [6,6-2H2] glucose enrichment during the insulin and glucose infusions. After a 2.5-h baseline period, an insulin infusion was started and continued for 4 h @ 40 mU/m^2^-min. Glucose was measured at 5-min intervals and maintained at 90 mg/dl. A 2 ml blood sample was collected at 0, 30, 60, 100, 110, and 120 min as well as every 10 min during the last 30 min of the clamp for GCMS determination of [6,6-2H2] glucose enrichment. Insulin and FFA samples were also drawn at multiple time points throughout the clamp. Skeletal muscle insulin sensitivity (R_d_/Insulin) was assessed as the rate of glucose disposal (mg/min) accounting for insulin during steady state. Hepatic insulin sensitivity was assessed as the suppression of endogenous glucose production (EGP) during steady state using the glucose enrichment data.

#### Blood Analyses

Lipid profiles (total cholesterol, HDL, LDL, VLDL, and triglycerides) and HbA1C were measured by a fasting blood draw and analyzed in the clinical chemistry laboratory at AH TRI using standard assays. Insulin resistance was also quantified using HOMA-I*R* = fasting plasma insulin (mU/L)^*^fasting plasma glucose (mg/dL)/405.

#### Muscle Biopsy

During fasting conditions and following 30–45 min after the start of the glucose clamp, a percutaneous muscle biopsy of the *vastus lateralis* was performed using previously published methods (11). A biopsy sample was taken 10–15 cm above the knee under local anesthesia with a 5-mm Bergstrom needle and suction. A portion of the tissue was prepared for immunohistochemistry.

Histochemical analyses were performed on serial sections using established methods in our laboratory (12). Briefly, muscle was placed vertically in mounting medium on cork and frozen in isopentane cooled with liquid nitrogen. Biopsy samples were sectioned (10 um) using a cryotome and fixed prior to staining. Sections were incubated in a primary antibody cocktail at 4°C overnight [BA-F8 (type I; IgG2b; 1:50); 6H1 (type IIX; IgM; 1:50); and SC-71 (type IIA; IgG1; 1:50)]. All antibodies were obtained from the University of Iowa Hybridoma Bank. Subsequently, slides were incubated in secondary antibody cocktail consisting of DyLight 405 (IgG2b; 1:500), Alexa Fluor 555 (IgM; 1:500), and Alexa Fluor 488 (IgG1; 1:500). AlexaFluor 647-conjugated wheat germ agglutinin (WGA) was used to stain glycoconjugate (N-acetylglucosamine and N-acetylneuraminic acid) residues. Digital images (4X magnification) of one section per skeletal muscle biopsy were captured using a Nikon eclipse Ti microscope (Nikon Technologies, California) and image analysis was performed using NIS elements software 4.20.01.

### Statistical Analysis

One-way ANOVAs were performed to evaluate baseline differences between groups. In cases where the assumption of normality (assessed using the Shapiro Wilk test) was not met, baseline comparisons between groups for these specific variables were performed using the non-parametric Kruskal Wallis test.

Two composite scores (C_Score) were developed to assess changes in insulin sensitivity measures and cardiometabolic risk factors using the average of standardized z scores. C_Scores for both outcomes were calculated using the following equation:

$$\begin{array}{l} \text{Composite Score}:\text{C}=\frac{z_{1}+z_{2}+z_{3}}{3}\\ \text{ Where }z_{i}=\frac{x_{i}-{\bar{x}}_{i}}{s_{i}},\\ \;{\bar{x}}_{i}\text{is the sample mean of variable i},\\ \;s_{i}\text{is the sample standard deviation of}\\ \text{ variable i}. \end{array}$$

The C_Score for insulin sensitivity (C_IS) comprises the % change in R_d_/Insulin from the clamp (*x* = % change), % change in HOMA-IR [*x* = –(% change)] and change in HbA1C [*x* = –(% change)]. The C_Score for cardiometabolic risk (C_CMR) comprises the % change in waist circumference [*x* = –(% change)], % change in triglycerides [*x* = –(% change)], and % change in fasting glucose [*x* = –(% change)]. The median of the composite scores was used to categorize participants as either “Low Responders” or “High Responders,” where High Responders had C_Score ≥ the median C_Score and conversely, Low Responders had C_Score < the median C_Score.

One-way ANOVAs were performed to assess between-group differences for C_IS and C_CMR. When a significant difference for the overall model was detected, a Tukey's *post-hoc* test for multiple comparisons was performed. To determine the effects of HED, WL and WL + EX on the proportion of individuals who were classified as “High Responder” and “Low Responder,” a 2 × 3 contingency table was generated, and group proportions were compared using the chi-square test. Because the WL and WL + EX groups were not matched for weight change, a one-way ANOVA was performed to assess between-group differences in weight change adjusted for baseline body weight. Additionally, regression analyses were run to assess the relationship between change in body weight and the composite scores, collapsed across the WL and WL + EX groups. Relationships between baseline values of participant characteristics and intervention-induced changes reflected by C_Scores were determined using Pearson correlation coefficients in both the WL and WL + EX groups. Statistical analysis was completed using GraphPad Prism version 8.1.2 for Windows (GraphPad Software, San Diego, California USA) and IBM SPSS Statistics for Macintosh, Version 25 (Armonk, NY:IBM Corp).

## Results

Participant baseline characteristics and ranges of percent change following intervention are summarized in Table 1. There were no significant differences in baseline characteristics between the HED, WL, and WL + EX groups.

**Table 1:** Baseline participant characteristics (mean ± SD) and range of change following intervention (%).

	**HED**		**WL**		**WL** **+** **EX**	
	**Baseline**	**Range of % change**	**Baseline**	**Range of % change**	**Baseline**	**Range of % change**
**n**	20		21		20	
Male:Female	7:13		7:14		8:12	
Age (yr)	70.1 ± 4.8		70.0 ± 4.6		66.8 ± 3.4	
**Medication use (no. of participants)**						
Statins	9		7		7	
Metformin	6		3		4	
Other Anti-hyperglycemic agents	1		2		1	
**Body composition**						
Weight (kg)	97.8 ± 10.5	−7.7 to 5.8	101.4 ± 20.3	−17.1 to 1.3	102.9 ± 13.2	−21.0 to (–)4.0
Body mass index (kg/m^2^)	35.7 ± 4.4	−7.8 to 5.5	36.1 ± 5.1	−16.5 to 1.3	37.3 ± 5.4	−17.4 to (–)3.5
Fat mass (kg)	44.7 ± 9.4	−14.8 to 8.8	46.7 ± 11.2	−36.3 to 5.9	47.1 ± 10.2	−37.5 to (–)4.5
Fat free mass (kg)	53.6 ± 5.9	−9.3 to 5.9	54.3 ± 12.3	−9.0 to 3.0	56.1 ± 9.5	−9.1 to 3.2
Waist circumference (cm)	114.9 ± 9.9	−10.1 to 6.6	116.4 ± 13.6	−16.1 to 20.0	118.5 ± 14.3	−16.7 to 5.4
Abdominal AT (kg)	20.7 ± 3.3	−14.0 to 16.5	21.2 ± 2.1	−20.6 to 0.2	22.7 ± 5.2	−28.4 to to (–)4.5
Abdominal subcutaneous AT (kg)	14.3 ± 2.6	−17.9 to 5.9	12.7 ± 2.6	−19.7 to 7.6	14.7 ± 3.9	−28.1 to (–)4.8
Abdominal visceral AT (kg)	6.4 ± 1.8	−24.8 to 58.8	8.4 ± 3.1	−23.2 to 16.1	8.0 ± 2.8	−37.0 to 4.7
Thigh intermuscular AT (kg)	0.38 ± 0.09	−13.1 to 42.4	0.42 ± 0.17	−14.8 to 17.9	0.48 ± 0.14	−33.0 to 5.2
**Clinical measurements**						
SBP (mmHg)	140 ± 11	−16.9 to 11.6	135 ± 15	−29.2 to 20.8	135 ± 11	−18.9 to 14.7
DBP (mmHg)	74 ± 8	−25.0 to 26.2	75 ± 11	−23.3 to 26.9	73 ± 12	−23.3 to 24.1
Insulin (pmol/l)	97.9 ± 46.5	−60.9 to 71.1	103.5 ± 68.8	−58.5 to 35.8	109.7 ± 55.6	−54.6 −54.6 to 46.9
Glucose (mmol/l)	6.0 ± 1.0	−28.8 to 78.6	5.5 ± 0.6	−0.8 to 0.2	6.1 ± 1.2	−37.9 to 6.9
HbA1C (%)	6.3 ± 0.8	−1.9 to 1.7	5.9 ± 0.4	−12.1 to 3.3	6.3 ± 0.9	−2.9 to 0.1
HOMA-IR	3.8 ± 2.5	−62.8 to 80.1	4.5 ± 3.5	−63.6 to 29.8	5.0 ± 3.7	−61.6 to 57.1
GIR/I (mg/kgFFM/min/Insulin)	0.08 ± 0.04	−53.2 to 100	0.08 ± 0.05	−40.5 to 196	0.07 ± 0.04	13.5 to 116
Triglycerides (mmol/l)	1.66 ± 0.63	−43.4 to 91.8	1.54 ± 0.76	−76.6 to 83.6	1.80 ± 0.76	−81.2 to 162
Cholesterol (mmol/l)	4.94 ± 0.98	−30.5 to 60.3	4.64 ± 0.95	−24.8 to 80.1	4.76 ± 0.98	−43.3 to 16.8
LDL-Cholesterol (mmol/l)	2.79 ± 0.85	−44.1 to 100	2.63 ± 0.86	−20.9 to 201	2.76 ± 0.85	−52.3 to 52.5
HDL-Cholesterol (mmol/l)	1.38 ± 0.44	−13.2 to 43.6	1.30 ± 0.38	−15 to 51.3	1.17 ± 0.19	−25.0 to 22.7
VLDL-Cholesterol (mmol/l)	0.77 ± 0.29	−44.8 to 90	0.71 ± 0.35	−77.0 to 83.3	0.83 ± 0.35	−81.5 to 170
Plasma free fatty acids (mmol/l)	0.47 ± 0.20	−93.1 to 885	0.53 ± 0.12	-42.4 to 33.1	0.56 ± 0.22	−74.8 to 216
**Aerobic fitness**						
VO_2max_ (L/min)	1.7 ± 0.5	−26.2 to 34.9	1.5 ± 0.5	−30.9 to 80.3	1.7 ± 0.5	−13.3 to 31.1
VO_2max_ (ml/kgFFM/min)	31.2 ± 7.4	−27.8 to 27.4	27.8 ± 6.9	−28.0 to 80.4	30.8 ± 4.6	−9.2 to 35.4
ATPmax	0.46 ± 0.13	−45.6 to 91.7	0.44 ± 0.09	−33.5 to 76.7	0.56 ± 0.23	−24.3 to 149
**One-step clamp (Values during steady state)**						
Suppression of FFA (%)	82.8 ± 25.9	−904 to 14.1	93.5 ± 5.8	−92.6 to 16.8	90.4 ± 10.0	−6.2 to 27.1
Suppression of EGP (%)	78.6 ± 20.8	−37.9 to 67.7	73.6 ± 11.6	−77.6 to 34.7	69.8 ± 25.2	−80.6 to 65.5
Rate of glucose disposal (mg/min/Insulin)	4.7 ± 2.1	−46.0 to 236	4.7 ± 2.2	−32.4 to 110	4.7 ± 2.5	−10.2 to 385
**Skeletal muscle histology**						
Type I fiber proportion (%)	40.4 ± 11.4	−27.1 to 47.7	41.8 ± 15.7	−38.5 to 28.9	38.9 ± 15.3	−27.7 to 36.6
Type IIA fiber proportion (%)	36.2 ± 13.5	−46.3 to 17.9	32.7 ± 13.3	−20.4 to 23.2	33.2 ± 12.0	−9.2 to 24.8
Type IIA/IIX fiber proportion (%)	8.4 ± 5.6	−7.1 to 5.6	6.7 ± 6.2	−10.1 to 11.2	6.9 ± 7.4	−29.8 to 14.1
Type IIX fiber proportion (%)	14.7 ± 13.1	−12.7 to 45.2	18.6 ± 13.5	−28.1 to 28.3	20.6 ± 13.2	−31.9 to 22.2
Type I CSA (μm^2^)	4,365 ± 991	−36.3 to 46.8	4,082 ± 939	−57.8 to 77.1	4,511 ± 1,322	−36.8 to 46.3
Type IIA CSA (μm^2^)	4,303 ± 1,503	−72.5 to 103	3,470 ± 848	−38.6 to 58.8	4,101 ± 1,276	−48.1 to 28.5
Type IIA/IIX CSA (μm^2^)	3,739 ± 1,520	−60.4 to 137	3,585 ± 1,899	−76.9 to 40.5	4,068 ± 2,211	−72.2 to 48.4
Type IIX CSA (μm^2^)	3,363 ± 1,718	−49.7 to 190	3,340 ± 1,339	−63.2 to 63.7	3,131 ± 1,294	−44.5 to 23.7
Capillary density (# capillaries/fiber CSA)	1.2 ± 0.5	−35.7 to 39.2	0.9 ± 0.4	−85.0 to 123	1.2 ± 0.6	−49.0 to 122

Range of change (%) = post-intervention − pre-intervention/pre-intervention*100%, with the exception of HbA1C, Suppression of FFA and EGP, and fiber type proportions, in which % range of change = post-intervention − pre-intervention.

*AT, adipose tissue; SBP, systolic blood pressure; DBP, diastolic blood pressure; HOMA-IR, Homeostatic Model Assessment of Insulin Resistance; TG, triglycerides; LDL, low-density lipoproteins; HDL, high-density lipoproteins; VLDL, very low-density lipoproteins; FFA, free fatty acids; GIR/I, Glucose Infusion Rate/Steady State Insulin; EGP, endogenous glucose production; CSA, cross-sectional area*.

*Sample size differs for the following characteristics*.

*Fasting Glucose: HED, n = 18; WL, n = 20; WL + EX, n = 19*.

*Abdominal AT, SAT, VAT, thigh IMAT: HED, n = 12; WL, n = 7; WL + EX, n = 13*.

*GIR/I, Fasting Insulin, HOMA-IR: HED, n = 19; WL, n = 13; WL + EX, n = 12*.

*Plasma FFA: HED, n = 18; WL, n = 17; WL + EX, n = 17*.

*VO_2max:_ HED, n = 20; WL, n = 19; WL + EX, n = 20*.

*ATPmax: HED, n = 13; WL, n = 8; WL + EX, n = 11*.

To ensure that the C_Scores were capturing a favorable change in insulin sensitivity and cardiometabolic risk, respectively, simple correlations between the C_Score and each of its components were assessed. C_IS was significantly correlated with an increase in R_d_/Insulin (*r* = 0.66, *p* < 0.0001), and a decrease in HOMA-IR (*r* = −0.82, *p* < 0.0001) and HbA1C (*r* = −0.62, *p* < 0.05). C_CMR was significantly correlated with a decrease in waist circumference (*r* = −0.65, *p* < 0.0001), fasting glucose (*r* = −0.68, *p* < 0.0001), and fasting triglycerides (*r* = −0.56, *p* < 0.001). Individual responses for each component of the C_Scores are illustrated in Supplementary Figures 1, 2.

**Figure 1:**
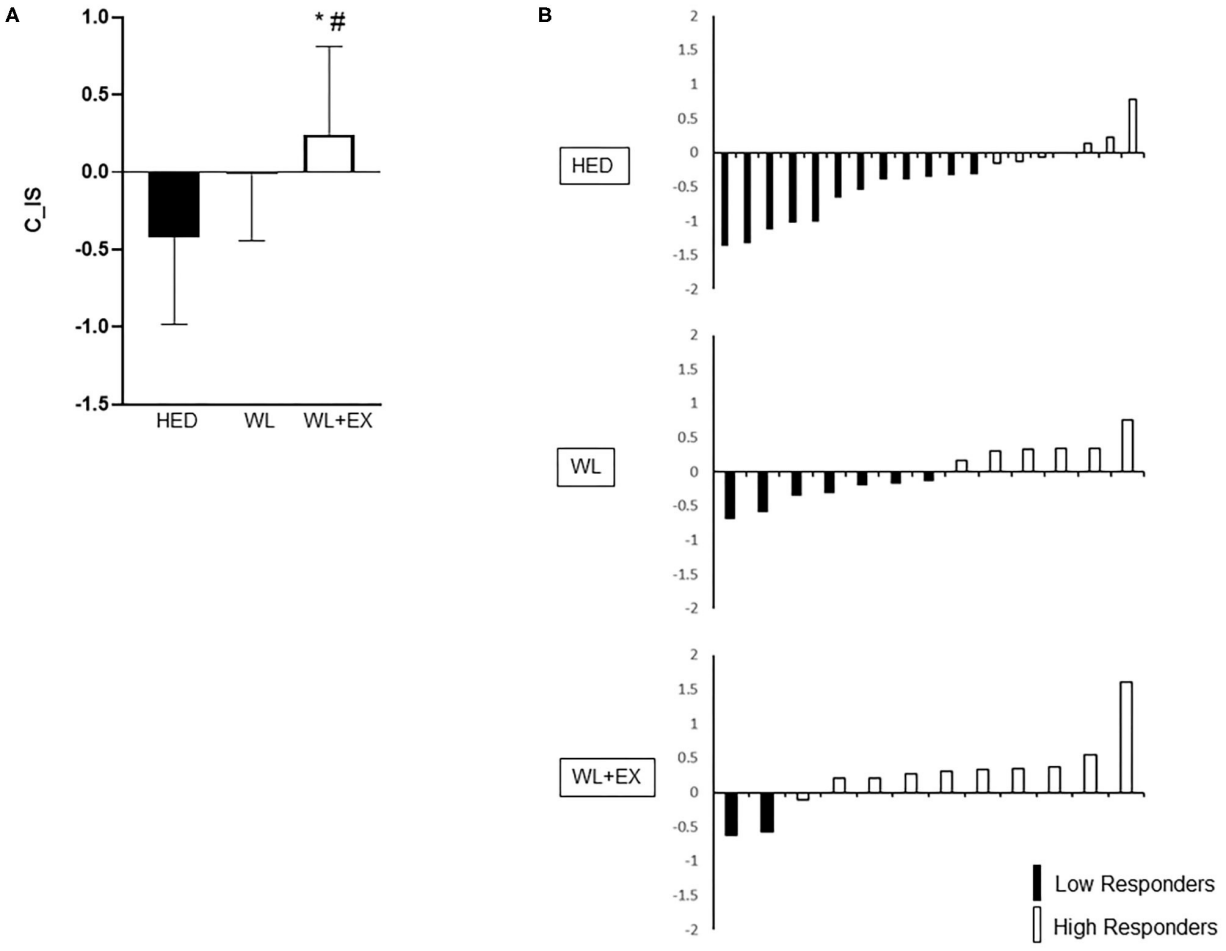
Group differences [**(A)**; mean C_IS and SD] and heterogeneity in individual response **(B)** for C_IS. *Significantly different from HED; ^#^Significantly different from WL.

**Figure 2:**
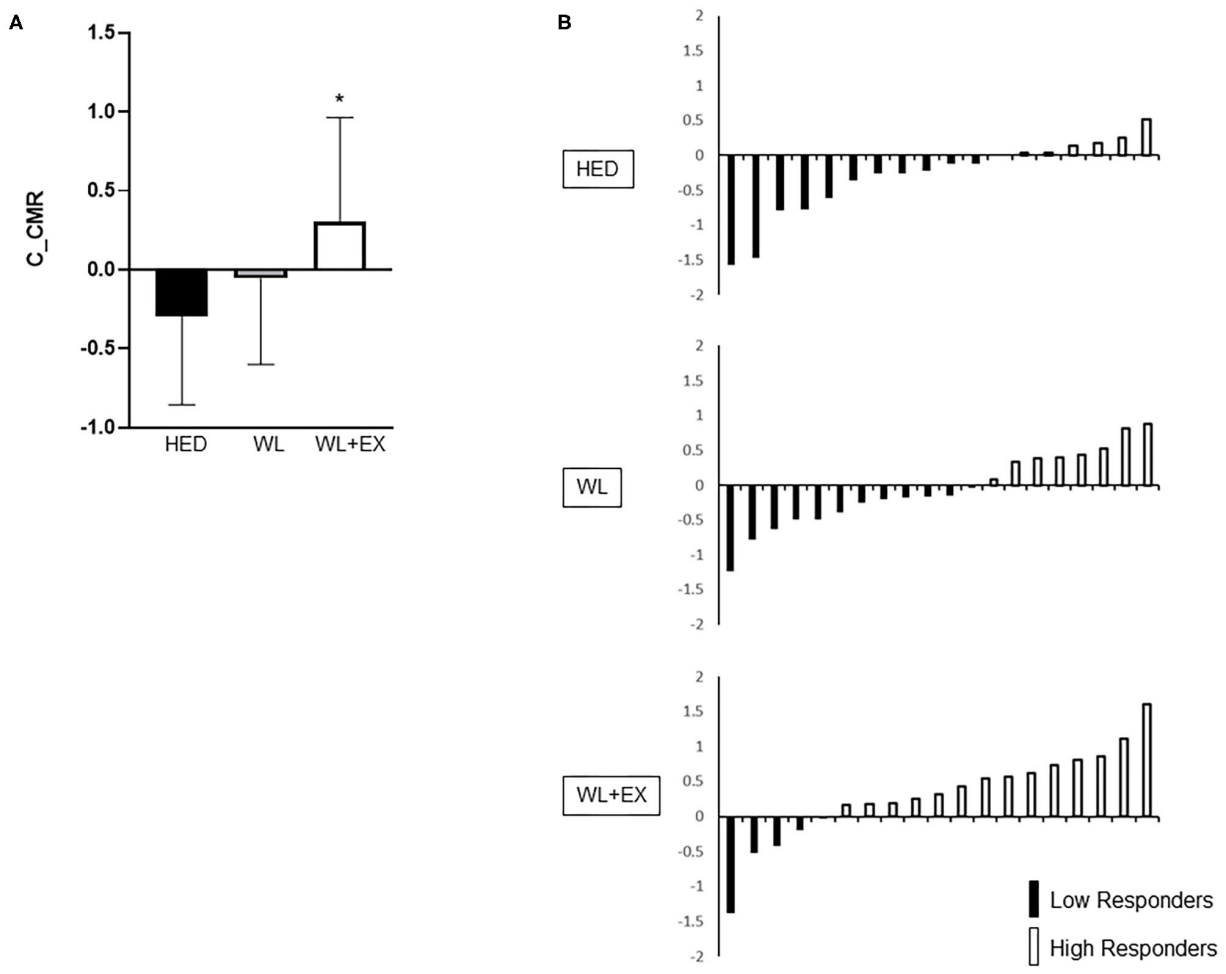
Group differences [**(A)**; mean C_CMR and SD] and heterogeneity in individual response **(B)** for C_CMR. *Significantly different from HED.

Between group comparisons for C_IS and C_CMR in addition to individual responses are shown in Figures 1, 2. The WL + EX group had a greater mean C_IS compared to both the WL and HED groups (*p* < 0.05, Figure 1A). The WL + EX group also had a greater mean C_CMR compared to the HED group (*p* < 0.05, Figure 2A), but not the WL group.

For C_IS, the WL and WL + EX groups had a greater proportion of High Responders (HR) compared to the HED group (HR proportions: HED = 32%, WL = 46%, WL + EX = 83%). In addition, the WL + EX group had a greater proportion of High Responders compared to the WL only group (*X*^2^ = 8.54, *p* = 0.014; Figure 1B). Similarly, for C_CMR, the WL + EX group had a greater proportion of High Responders compared to both the WL and HED groups (HR proportions: HED = 39%, WL = 40%, WL + EX = 74%) (*X*^2^ = 6.12, *p* < 0.05; Figure 2B). The WL and WL + EX groups differed in body weight change (WL vs. WL + EX: −7.1 ± 4.6 kg vs. −10.6 ± 4.9 kg; *p* < 0.05). C_IS (*r* = −0.42; *p* < 0.05), but not C_CMR (*p* > 0.05), was significantly associated with weight change.

Associations between C_Scores and baseline characteristics, including body composition, clinical laboratory measures, aerobic fitness, insulin sensitivity, and fiber type are summarized in Table 2. For change in insulin sensitivity (C_IS), pre-intervention measures of insulin (*r* = 0.60) and HOMA-IR (*r* = 0.56) were positively associated with C_IS in the WL group only (*p* < 0.05). For change in cardiometabolic risk (C_CMR) glucose (*r* = 0.55), triglycerides (*r* = 0.53), and VLDL (*r* = 0.53) were positively associated with C_CMR in the WL only group (all *p* < 0.05). In the WL + EX group, baseline triglycerides (*r* = 0.59) and VLDL (*r* = 0.59) were positively associated with C_CMR (all *p* < 0.05).

**Table 2:** Associations between baseline characteristics and C_Scores.

**Characteristic**	**WL**		**WL** **+** **EX**	
	**C_IS (r)**	**C_CMR (r)**	**C_IS (r)**	**C_CMR (r)**
**Age**	−0.36	−0.09	0.22	0.11
**Body composition**				
Weight	−0.27	−0.32	−0.16	−0.19
Body mass index	−0.23	−0.33	0.00	0.04
Waist circumference	−0.37	−0.27	−0.07	0.10
Abdominal AT	−0.04	−0.16	−0.15	−0.26
Abdominal subcutaneous AT	0.12	−0.69	−0.28	−0.13
Abdominal visceral AT	−0.12	0.48	0.08	−0.30
Thigh intermuscular AT	−0.33	0.51	0.66	0.04
**Clinical measurements**				
SBP	0.25	0.33	−0.22	−0.25
DBP	0.14	0.16	−0.50	−0.39
Insulin	0.58^*^	−0.21	0.41	−0.06
Glucose	0.26	0.55^*^	0.10	0.36
HbA1C	−0.17	−0.25	0.56	0.37
HOMA-IR	0.57^*^	−0.14	0.31	−0.09
Triglycerides	0.33	0.53^*^	0.23	0.59^*^
Cholesterol	0.46	0.15	−0.13	0.15
LDL-Cholesterol	0.45	0.06	−0.19	−0.07
HDL-Cholesterol	−0.07	−0.27	0.03	0.16
VLDL-Cholesterol	0.33	0.53^*^	0.22	0.59^*^
Plasma free fatty acids	0.78^*^	0.08	0.18	0.07
**Aerobic fitness**				
VO_2max_ (l/min)	0.16	0.09	−0.08	−0.30
VO_2max_ (ml/kgFFM/min)	0.19	0.25	0.10	−0.06
ATPmax	−0.33	−0.30	−0.14	0.22
**One-step clamp (Values during steady state)**				
GIR/I	−0.50	−0.15	−0.44	0.06
Suppression of FFA	−0.02	0.05	0.22	−0.29
Suppression of EGP	−0.04	0.41	0.32	0.44
Rate of glucose disposal	–0.59^*^	−0.04	–0.60^*^	−0.06
**Skeletal muscle fiber type**				
Type I fiber proportion	−0.28	0.29	−0.52	0.01
Type IIA fiber proportion	−0.03	−0.11	−0.07	0.32
Type IIA/IIX fiber proportion	0.44	−0.24	−0.04	−0.18
Type IIX fiber proportion	0.15	−0.12	0.64	−0.11
Type I CSA	0.17	0.35	0.64	0.18
Type IIA CSA	−0.01	0.16	0.50	−0.15
Type IIA/IIX CSA	−0.42	0.15	0.40	−0.10
Type IIX CSA	0.02	0.27	0.33	−0.21
Capillary density	0.24	0.43	−0.38	−0.08

*AT, adipose tissue; SBP, systolic blood pressure; DBP, diastolic blood pressure; HOMA-IR, Homeostatic Model Assessment of Insulin Resistance; TG, triglycerides; LDL, low-density lipoproteins; HDL, high-density lipoproteins; VLDL, very low-density lipoproteins; FFA, free fatty acids; GIR/I, Glucose Infusion Rate/Steady State Insulin; EGP, endogenous glucose production; CSA, cross-sectional area; WL, weight loss; WLEX, weight loss with exercise; C_IS, insulin sensitivity composite score; C_CMR, cardiometabolic risk composite score*.

* *Bold indicates significant association between baseline characteristic value and C_Score at p < 0.05*.

## Discussion

Recent focus on the application of personalized lifestyle-based medicine in the last decade has stimulated an exponential increase in observations related to response heterogeneity. However, several questions remain including the relative effect of different types of lifestyle-based prescriptions (exercise and/or diet) on interindividual variability, particularly in vulnerable populations such as older obese adults. In the present study, our primary findings indicate that the addition of exercise to energy restriction-induced weight loss improves the proportion of High Responders for glycemic control and cardiometabolic risk compared to weight loss alone and a time-matched control group. Our findings have novel implications for enhancing our understanding of the impact of lifestyle interventions on the variability of important clinical variables in older obese adults that may support future efforts to tailor lifestyle interventions to the individual and optimize treatment outcomes.

To our knowledge no prior studies have assessed the independent contributions of weight loss with or without exercise to the response heterogeneity in insulin sensitivity and cardiometabolic risk, particularly in the older obese population. Additionally, in prior analyses that examine variability, studies have typically been small, and the majority lack a control group, precluding the ability to assess intervention-independent effects on response (4, 5, 13). The current trial includes a time-matched control group that allows assessment of intervention responses beyond both technical error and day-to-day biological fluctuations (6, 7). Using this approach, we observed that exercise combined with energy intake restriction-induced weight loss is a superior approach for improving the proportion of individuals who achieve a favorable response for both insulin sensitivity and cardiometabolic risk compared to weight loss alone or no intervention. While others have suggested a similar mean group response to exercise vs. diet-induced weight loss in men for several clinical outcomes (14–17), our findings suggest that more individuals will achieve a greater response magnitude to intervention with the combination of diet-induced weight loss and exercise compared to diet alone. Taken together, our novel findings reinforce and provide support for the inclusion of regular exercise in addition to dietary recommendations to improve the likelihood that an individual responds favorably to treatment.

We completed a comprehensive assessment of relationships between baseline traits and response for glycemic control and cardiometabolic risk, including clinical laboratory outcomes, MRI-derived body composition, aerobic fitness, muscle and hepatic insulin sensitivity, and immunohistochemical analysis of fiber type and capillary density. Overall, while pre-intervention traits were differentially associated with insulin sensitivity and cardiometabolic risk response, in both WL and WL + EX groups a more favorable response to intervention was associated with a higher risk clinical phenotype at baseline. Specifically, in both intervention groups, higher baseline triglycerides and VLDL-cholesterol were associated with greater improvement in cardiometabolic risk while higher plasma insulin and HOMA-IR were associated with increased insulin sensitivity. Consistent with our findings are those from a 12-week diet and exercise intervention in individuals aged 18–75 years who were at risk for type 2 diabetes (9), wherein High Responders for glucose AUC assessed by 2-h OGTT had higher baseline weight, visceral AT, fasting glucose, 2-h OGTT glucose, and triglycerides and lower HDL-cholesterol compared to those who experienced an adverse response or attenuated response to the intervention. However, our findings also contradict many others who observed blunted responses to exercise interventions associated with metabolically unhealthy outcome levels at baseline (4, 5, 18–20). Several factors may explain the discrepant findings, including differences in sample demographics and disease diagnosis, duration of disease, medication use, dissimilar outcome variables, correlation vs. categorical response analysis, intervention characteristics, etc. Thus, further investigation is warranted to evaluate whether response heterogeneity and predictors of response differ across population subtypes and lifestyle modifications to move closer to personalized lifestyle medicine that optimizes changes in clinical outcomes based on individual characteristics.

Numerous mechanisms have been highlighted as potential contributors to an individual's response to lifestyle intervention (21, 22). Prior work from our group demonstrated that skeletal muscle DNA methylation and RNA expression patterns reflective of elevations in antioxidant defense, insulin signaling, and mitochondrial metabolism were present in Non-Responders based on changes in PCR recovery rate (i.e., *in vivo* muscle mitochondrial function) and insulin sensitivity following a 10-week aerobic exercise intervention (23). These molecular characteristics of Non-Responders correlated with higher baseline insulin sensitivity and muscle mitochondrial function *in vivo* (23). Taken together, these mechanistic findings support the interpretation of our observations that indicate a higher metabolic burden and less healthy skeletal muscle phenotype allows for a greater window of opportunity for improvement. Thus, factors across a range of molecular and metabolic outcomes (genetics, epigenetics, metabolism, physiology, etc.) likely play a role in an individual's response to intervention and should be further exploited in future studies (24).

Given growing interest in the study of individual responses and its implications for personalized exercise and diet prescription, it is important to consider the clinical relevance and interpretation of our findings. This notion is complicated by the range of important health outcomes under interrogation that do not necessarily change in concert. The use of Z-scores to reflect the concurrent change in a collection of predefined outcomes is not a novel concept (25–28). However, we extend this application to the study of interindividual variability. Compared to the interventions described above that focus on a singular outcome, the use of Z-scores appears to reduce the proportion of individuals who respond poorly or do not respond to intervention (24). Classifying an individual as a “non-responder” based on change in a singular outcome without consideration for equally meaningful changes in other outcomes may discourage these individuals from implementing positive exercise and dietary habits into their habitual routines. Furthermore, the use of Z-scores for predefined clusters of variables reduces the biological variability of each component (Supplementary Figures 1, 2), thus reducing the “noise” and more robustly capturing the response to the intervention itself (29). Thus, in this field of response heterogeneity, it may be helpful to consolidate related outcomes to provide an integrative assessment of physiological responses and improve clinical applications and inferences.

There are limitations in our study that should be considered. For some outcome variables, the sample size may not be adequate to assess associations between baseline phenotype and response to WL or WL + EX. This is particularly true for measures of skeletal muscle fiber type and MRI-derived AT. While these are simple associations and do not imply causation, our findings do prompt future work with appropriately powered trials to combine data from molecular, metabolic, physiological and clinical measures to assess predictors of response to weight loss with and without exercise. Our participants reflected a range in diabetes status, from no diabetes to frank type 2 diabetes and thus, differed in medication use. Recent interest in the interaction effects of exercise and medication use on response across a range of outcomes has revealed inconsistent findings. Observations in a large sample (*n* = 225) of men and women with type 2 diabetes suggest that metformin had no effect on HbA1C reduction following aerobic exercise training (30). Contrary to these findings, in both older adults (31) and those with prediabetes (32), the increase in whole-body insulin sensitivity following 12 weeks of aerobic exercise training was attenuated in those taking metformin concurrently. Similar discrepancies are seen with the interaction between statin use and exercise, where evidence from obese elderly males suggests no impact of statins on the beneficial effects of 12 weeks of exercise (33), whereas the addition of statins blunted the increase in cardiorespiratory fitness and citrate synthase activity in overweight or obese adults (34). Thus, just as individuals respond differently to exercise training, the interaction of his/her medication use with exercise may also differ. Taken together, factors associated with medication use (e.g., length of use, sex differences, disease status) may introduce variability in the response to exercise training for cardiometabolic risk and glycemic control. The WL and WL + EX groups were not matched for weight loss and we did not include an EX only group; thus, it is uncertain whether the improved response with exercise is a result of differences in energy balance or exercise *per se*. Additionally, we do not have adherence and compliance records for all participants; both may impact response variability. Future work carefully accounting for energy balance is warranted in order to definitively make these conclusions.

In conclusion, the addition of exercise to energy restriction-induced weight loss improves the number of older obese adults who achieve improvement in insulin sensitivity and cardiometabolic risk. Additionally, individuals with poorer metabolic status at baseline are more likely to experience greater improvements in clinical outcomes with these lifestyle interventions. Our data contributes novel findings with regards to individual response variation to lifestyle interventions, moving us closer to identifying predictors of response and tailoring lifestyle-based treatments to the individual.

## Data Availability Statement

The raw data supporting the conclusions of this article will be made available by the authors, without undue reservation.

## Ethics Statement

The studies involving human participants were reviewed and approved by Institutional Review Board of AdventHealth. The patients/participants provided their written informed consent to participate in this study.

## Author Contributions

BG concepted and designed the primary trial and assisted LS and AB in conceptualizing this secondary analysis. RS and EC coordinated the primary trial and organized all data collection. FY provided statistical support for the manuscript. AB completed statistical analysis and data interpretation. BG, LS, and AB were responsible for drafting the manuscript. All authors assisted with manuscript revision.

## Conflict of Interest

The authors declare that the research was conducted in the absence of any commercial or financial relationships that could be construed as a potential conflict of interest.
