# Supplementary material for: Individual Response Variation in the Effects of Weight Loss and Exercise on Insulin Sensitivity and Cardiometabolic Risk in Older Adults
Source: Front Endocrinol (Lausanne). 2020 Sep 10;11:632. doi: 10.3389/fendo.2020.00632 (PMC7511700; doi:10.3389/fendo.2020.00632)
Supplement: Supplementary file 2 [file Presentation_2.PPTX]

## Slide 1
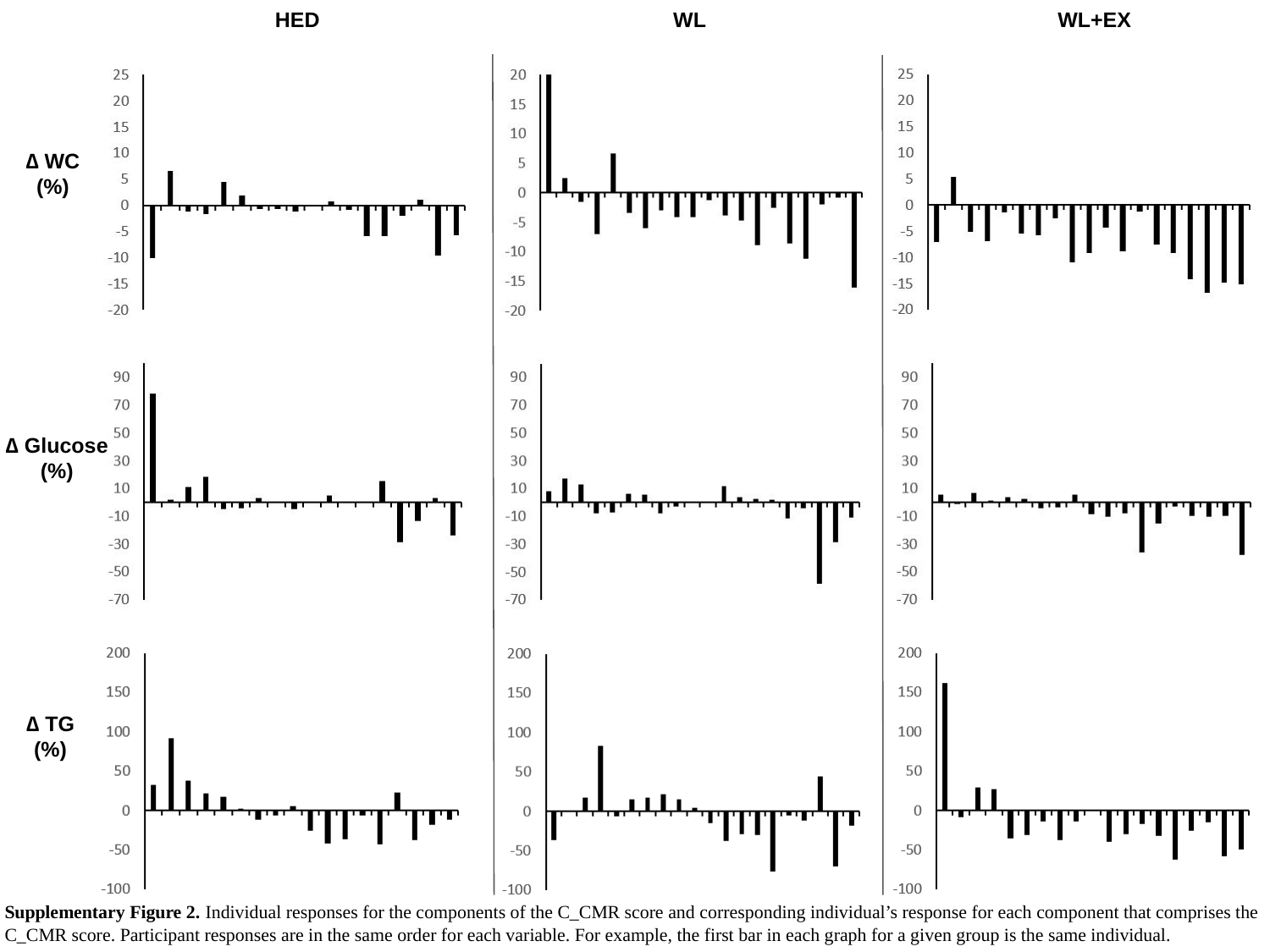

HED
WL
WL+EX
∆ WC
(%)
∆ Glucose
(%)
∆ TG
(%)
Supplementary Figure 2. Individual responses for the components of the C_CMR score and corresponding individual’s response for each component that comprises the C_CMR score. Participant responses are in the same order for each variable. For example, the first bar in each graph for a given group is the same individual.
